# Interface Enhancement and Tribological Properties of Cattle Manure-Derived Corn Stalk Fibers for Friction Materials: The Role of Silane Treatment Concentration

**DOI:** 10.3390/polym17010022

**Published:** 2024-12-26

**Authors:** Siyang Wu, Lixing Ren, Xiaochun Qiu, Qiance Qi, Bo Li, Peijie Xu, Mingzhuo Guo, Jiale Zhao

**Affiliations:** 1 College of Engineering and Technology, Jilin Agricultural University, Changchun 130118, China; siyangwu@outlook.com (S.W.); renlixingjlag@163.com (L.R.); qiuxiaochun0131@163.com (X.Q.); qianceqi774@outlook.com (Q.Q.); lboxxx@163.com (B.L.); pj15137475091@outlook.com (P.X.); 2 College of Biological and Agricultural Engineering, Jilin University, Changchun 130022, China

**Keywords:** biological pre-treatment, cattle manure, corn stalk fiber, interface modification, friction materials, tribological properties

## Abstract

Corn stalk fibers extracted from cattle manure (CSFCM) represent a unique class of natural fibers that undergo biological pre-treatment during ruminant digestion. This study systematically investigates the optimization of CSFCM-reinforced friction materials through controlled silane treatment (2–10 wt.%). The biological pre-treatment through ruminant digestion creates distinctive fiber properties that influence subsequent chemical modification. Physical characterization revealed that optimized interface modification at 6 wt.% silane treatment (CSFCM-3) effectively enhanced the fiber–matrix compatibility while achieving a 34.2% reduction in water absorption and decreased apparent porosity from 9.03% to 7.85%. Tribological evaluation demonstrated superior performance stability, with CSFCM-3 maintaining friction coefficients of 0.35–0.45 across 100–350 °C and exhibiting enhanced thermal stability through a fade ratio of 14.48% and recovery ratio of 95%. The total wear rate showed significant improvement, reducing by 26.26% to 3.433 × 10^−7^ cm^3^ (N·m)^−1^ compared to untreated specimens. Microscopic analysis confirmed that the optimized silane modification promoted the formation of stable secondary plateaus and uniform wear patterns, contributing to enhanced tribological performance. This investigation establishes an effective approach for developing high-performance friction materials through precise control of silane treatment parameters. The findings demonstrate the potential for developing sustainable friction materials with enhanced performance characteristics, offering new pathways for eco-friendly material design that effectively utilizes agricultural waste resources.

## 1. Introduction

The accelerating pace of industrial development has led to an overwhelming dependence on synthetic polymers, creating severe environmental challenges. Global plastic production has surged to approximately 400 million tons annually, with only 9% being recycled while the remainder accumulates in landfills and oceans [1], persisting for hundreds of years and gradually degrading into microplastics that contaminate ecosystems. To address these mounting environmental concerns, eco-friendly polymer-based composites have emerged as a promising solution, offering the potential to maintain desired performance characteristics while reducing environmental impact through the incorporation of sustainable reinforcing materials [2,3]. Natural fiber composites demonstrate significant environmental advantages, requiring 17% less energy in manufacturing and enabling higher energy recovery during end-of-life incineration (12–34 MJ/kg compared to 7.5 MJ/kg for glass fiber composites). Particularly in the automotive industry, where natural fiber composites have been widely adopted for interior components and structural parts, these materials have achieved weight reductions of up to 25% compared to conventional composites, contributing to improved fuel efficiency and reduced emissions [4,5]. The selection of appropriate reinforcement materials plays a crucial role in determining the overall properties and environmental impact of these composites. Among various reinforcement options, natural fibers have gained significant attention due to their biodegradability, renewability, abundance, and cost-effectiveness [6].

Among diverse natural fiber sources, corn stalk fibers have emerged as a promising reinforcement for composite materials. Corn stalk is a significant renewable resource, and among its various utilization pathways, feed use is one of the primary consumption routes, accounting for approximately 19% of the total corn stalk resources [7]. Ruminants, particularly cattle, can partially digest corn stalks, but a considerable amount of undigested stalk fibers remains in their manure. It is estimated that each cattle produces around 7.3 kg of dry manure per day, and in the United States alone, the annual dry cattle manure generation reaches about 24 billion tons [8]. Assuming that the corn stalk content in cattle manure is approximately 20%, the amount of recoverable corn stalk fibers from this waste stream would be substantial.

Corn stalk fibers extracted from cattle manure (CSFCM) have emerged as particularly promising due to their distinctive characteristics. These fibers undergo an inherent biological processing through the ruminant digestive system, where complex microbial ecosystems and specific physiological conditions facilitate selective degradation of plant fiber components. During ruminant digestion, the corn stalk fibers experience a series of physical, chemical, and biological modifications. The mechanical breakdown through rumination and muscular contractions, combined with exposure to rumen fluid (pH 6.0–6.8), initiates the degradation process [9]. The diverse microbial communities in the rumen, including bacteria, protozoa, and fungi, selectively degrade plant components such as hemicellulose and pectin while preserving the fundamental cellulose structure. This natural biological pre-treatment effectively removes non-structural components, resulting in enhanced cellulose content and modified fiber architecture. The rumen environment’s unique combination of mechanical actions, enzymatic processes, and microbial activities establishes favorable properties for composite material applications, providing an environmentally friendly alternative to conventional chemical pre-treatments [10].

The development of natural fiber-reinforced composites, particularly those utilizing CSFCM, represents a promising solution to address the growing plastic pollution crisis. These materials offer inherent biodegradability while maintaining comparable performance characteristics for specific applications, thereby reducing the accumulation of non-biodegradable waste in the environment. Furthermore, CSFCM-based composites create a dual environmental benefit by both reducing plastic waste through biodegradable alternatives and utilizing agricultural waste streams. The biological pre-treatment through ruminant digestion not only enhances fiber properties but also provides an environmentally friendly processing method. This approach aligns with circular economy principles and global initiatives to reduce plastic pollution, offering a practical pathway for industries to transition from petroleum-based materials to eco-friendly alternatives.

Recent studies have demonstrated the versatility and effectiveness of CSFCM across various applications. In cementitious composites, Li et al. [11] and Yang et al. [12] revealed that pre-treated cow dung fibers significantly improved mechanical properties and reduced shrinkage behavior. Yang et al. [13] successfully extracted cellulose fibers from cow dung waste and characterized their functional properties, demonstrating their potential in sustainable material development. In the field of friction materials, Ma et al. [14] reported excellent tribological and mechanical properties of cow dung fiber-reinforced friction composites, while Wu et al. [15] conducted comprehensive comparative studies showing competitive performance against traditional natural fibers like corn stalk and sisal. Thermal characteristics studies by Reddy et al. [16] confirmed the material’s stability at elevated temperatures. Moreover, recent research has expanded into specialized applications, including the development of cow dung-based paper [17], and alkali-activated slag mortars [18], and comprehensive reviews by Fasake and Dashora [19,20] have highlighted the sustainable potential of these materials across various industrial applications.

Despite the inherent advantages of CSFCM, significant technical challenges persist in developing high-performance natural fiber-reinforced composite materials, primarily concerning interface optimization. The fundamental challenge originates from the inherent incompatibility between hydrophilic functional groups present on natural fiber surfaces and hydrophobic polymer matrices, resulting in suboptimal stress transfer at the interface. Various surface modification methods have been developed to improve the compatibility between natural fibers and polymer matrices. Chemical treatments such as alkali treatment with NaOH effectively remove non-cellulosic components but may cause excessive fiber damage and cellulose depolymerization, particularly at concentrations above 5% [21]. Similarly, acetylation using acetic anhydride can reduce fiber hydrophilicity but risks fiber embrittlement and generates chemical waste [22]. Conventional biological pre-treatments like fungal degradation require controlled laboratory conditions and extended processing times [23]. In comparison, ruminant digestive processing presents a unique biological approach with several advantages. The ruminant digestive system provides natural pH regulation (6.0–7.0) and temperature control (38–42 °C) through physiological mechanisms, enabling selective degradation of hemicellulose and lignin while preserving cellulose structure [9]. This process is environmentally sustainable and generates no chemical waste. However, challenges include seasonal variations in fiber quality and the need for post-extraction purification. Despite these limitations, the controlled biological modification through ruminant digestion offers a promising alternative for natural fiber pre-treatment.

Among these surface modification approaches, silane treatment has emerged as an effective complementary method to biological pre-treatment. While ruminant digestion effectively removes non-cellulosic components and creates active sites on fiber surfaces, subsequent silane coupling treatment can further enhance fiber–matrix interfacial bonding. The silane coupling agent (KH-550) creates chemical bridges between the hydrophilic fiber surface and hydrophobic polymer matrix through its bifunctional molecular structure. This combined approach of biological pre-treatment followed by silane modification potentially offers synergistic benefits: the biological process provides a “green” initial modification while silane treatment ensures strong interfacial adhesion in the final composite.

This research presents a comprehensive investigation of CSFCM-reinforced polymer composites, focusing on silane treatment parameter optimization and subsequent effects on friction performance. The methodology systematically examines varying silane concentrations (2–10 wt.%) on fiber properties and composite characteristics. Biological pre-treatment, particularly the fiber extraction from cattle manure in this study, achieves a secondary utilization of traditional biomass resources and enhances their application value. The combination of cattle manure fiber extraction with silane treatment further improves the mechanical properties of cattle manure straw fiber-reinforced materials. This combined approach has shown particular promise in tribological applications. The research methodology and findings from this study provide valuable insights for expanding the potential applications of these modified fibers in other fields.

## 2. Materials and Methods

### 2.1. Materials

#### 2.1.1. Raw Materials and Formulation

The raw materials for this investigation were systematically selected based on their functional roles in the composite system. Corn stalk fibers extracted from cattle manure (CSFCM) were obtained from a Simmental cattle breeding farm (Changchun, China) as the primary fibrous reinforcement. A commercial phenolic resin (Jinan Shengquan Group Share-Holding Co., Ltd., Jinan, China) served as the matrix binder. The modification system comprised multiple functional components: graphite (Qingdao Matery Graphite Co., Ltd., Qingdao, China), antimony sulfide (Xiamen Ditai Chemicals Co., Ltd., Xiamen, China), bauxite (Zhengzhou Highland Refractory Material Co., Ltd., Zhengzhou, China), zinc stearate (Nanjing Lepuz Chemical Co., Ltd., Nanjing, China), porous iron powder (Shanghai Knowhow Powder-Tech Co., Ltd., Shanghai, China), and petroleum coke (Changge Youtong Environmental Protection Materials Co., Ltd., Changge, China). Additional reinforcement was provided by compound mineral fibers (Hebei Runhuabang New Material Technology Co., Ltd., Shijiazhuang, China). The filler system consisted of vermiculite powder (Hebei Ansta Trading Ltd., Shijiazhuang, China), barium sulfate, and friction powder (Guangzhou Billion Peak Chemical Technology Co., Ltd., Guangzhou, China). KH-550 silane coupling agent (Dalian Richon Chem Co., Ltd., Dalian, China) was employed for interface optimization. The precise compositional ratios are detailed in Table 1.

#### 2.1.2. Fiber Extraction and Treatment

**Figure 1:**
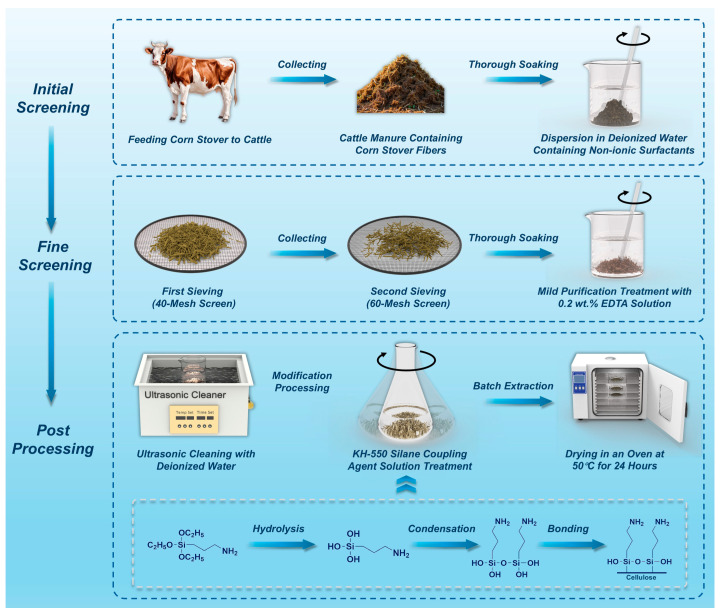
Schematic illustration of the three-stage extraction and modification process for corn stalk fibers from cattle manure (CSFCM): initial screening, fine screening, and post-processing stages.

The extraction and modification of corn stalk fibers from cattle manure (CSFCM) was systematically executed through a precisely controlled three-stage process, as illustrated in Figure 1. In the initial screening stage, corn stover was first fed to cattle at a local Simmental breeding farm in Changchun, China. The fresh cattle manure containing partially digested corn stover fibers was collected within 24 h of excretion to maintain optimal fiber conditions. The collected manure was immediately dispersed in deionized water (resistivity > 18.2 MΩ·cm) containing 0.5 wt.% non-ionic surfactants at a material/liquid ratio of 1:5 (*w*/*v*). The dispersion process was maintained at 30 ± 2 °C for 2 h under continuous stirring to ensure thorough fiber separation and preliminary purification.

In the fine screening stage, the dispersed material underwent a precise two-step sieving process. The first screening was conducted using a standardized 40-mesh stainless-steel sieve (aperture size: 420 μm) under controlled water flow (2 L/min) to remove coarse impurities. The filtered material was then passed through a finer 60-mesh screen (aperture size: 250 μm) under the same flow conditions for more precise fiber separation. The retained fibers underwent a mild purification treatment in 0.2 wt.% EDTA solution (pH 7.5 ± 0.2) at 25 ± 1 °C for 60 min with gentle agitation at 10 rpm to chelate and remove metallic impurities while maintaining the structural integrity of the fibers.

The post-processing stage comprised three critical steps. Firstly, the fibers were subjected to ultrasonic cleaning in deionized water for 30 min at 25 ± 1 °C. Second, the cleaned fibers underwent surface modification with pre-hydrolyzed KH-550 silane coupling agent. The coupling agent solution preparation followed a strictly controlled protocol: KH-550 was hydrolyzed in an ethanol/water mixture (95:5 *v*/*v*, HPLC grade ethanol, purity ≥ 99.9%) under controlled pH (4.0 ± 0.2, adjusted with glacial acetic acid) and temperature (25 ± 0.5 °C) conditions. The hydrolyzed solution was then precisely diluted to target concentrations (2, 4, 6, 8, and 10 wt.%) using Class A volumetric glassware, with concentration verification through specific gravity measurements (±0.0002 g/cm^3^) at 25 °C. The surface modification was conducted at 25 ± 1 °C for 120 min under controlled stirring (200 ± 5 rpm), with solution stability maintained through 4 h renewal intervals and regular pH verification. Finally, the modified fibers were dried in a programmable vacuum oven (LC-DZF-6020AB, Shanghai Lichen-BX Instrument Technology Co., Ltd., Shanghai, China) at 50 ± 0.5 °C under −0.08 MPa pressure for 24 h to achieve a final moisture content below 2 wt.%.

KH-550 serves as an effective silane coupling agent to enhance the interfacial adhesion between natural fibers and polymer matrices. The chemical interaction between KH-550 and cellulosic fibers involves a series of hydrolysis and condensation reactions. Initially, the ethoxy groups (-OC_2_H_5_) of KH-550 undergo hydrolysis in the presence of moisture, converting to reactive silanol groups (-Si-OH). Subsequently, these silanol groups can form stable covalent bonds (-Si-O-Cellulose) with the abundant hydroxyl groups (-OH) on the surface of the cellulosic fibers via a condensation reaction [24]. This chemical bonding mechanism significantly improves fiber–matrix interfacial adhesion, resulting in the enhanced mechanical properties and durability of the natural fiber-reinforced polymer composites. The grafting of KH-550 onto cellulosic fibers not only increases the compatibility between the hydrophilic fibers and the hydrophobic polymer matrix but also provides a strong and stable link at the interface. This interfacial modification promotes efficient stress transfer from the matrix to the reinforcing fibers, ultimately leading to the improved overall performance of the composite materials. Furthermore, the amine groups (-NH_2_) in the KH-550 molecules can chemically react with the resin matrix, thereby creating a “molecular bridge” between the cellulosic fibers and polymer matrix. This serves to improve the compatibility and stress transfer efficiency of the composite system. Through these chemical reactions, KH-550 forms an amine-containing coating layer on the surface of the cellulosic fibers, which enhances the wettability and interfacial bonding strength between the hydrophilic natural fibers and hydrophobic polymer matrix.

In summary, the bifunctional nature of KH-550 enables it to chemically bond with both the hydroxyl groups on the cellulosic fiber surface and the functional groups in the polymer resin, resulting in markedly improved fiber–matrix interfacial adhesion. This surface modification method using silane coupling agents has been widely applied in natural fiber-reinforced polymer composites to enhance their mechanical properties and durability.

#### 2.1.3. Preparation of Friction Materials

**Figure 2:**
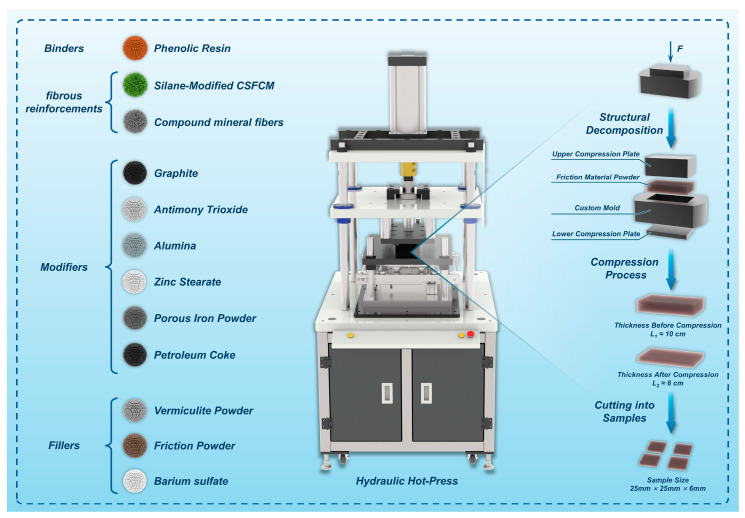
Schematic diagram of the hot-press molding principle for friction materials.

The friction materials were fabricated using a precision-controlled hot-press molding system (JFY500, Jilin University Electromechanical Equipment Co., Ltd., Changchun, China). As illustrated in Figure 2, the molding system utilized custom-designed cast iron molds positioned between the upper and lower hot-press platens. The material consolidation process was precisely regulated through three critical parameters: molding temperature, pressure, and dwelling time. The following optimal processing parameters were established through systematic optimization studies: molding temperature of 160 °C, compression pressure of 45 MPa, and dwelling time of 30 min. The hot-press mold assembly was preheated to 150–160 °C prior to material loading to ensure uniform heat distribution.

To mitigate potential defects associated with volatile evolution during the curing process, which could manifest as surface blistering, swelling, or microcracking, a programmed degassing protocol was implemented. The protocol consisted of three sequential cycles of 10 s compression followed by 12 s degassing before transitioning to sustained pressure application. The consolidated specimens underwent controlled post-cure treatment to ensure complete phenolic resin crosslinking and minimize residual stress-induced dimensional instability. Subsequently, the materials were machined to precise dimensions (25 mm × 25 mm × 6 mm) through surface grinding and cutting operations for characterization studies.

The samples were systematically designated according to the silane coupling agent concentration gradient as follows: CSFCM-0 (unmodified control), CSFCM-1 (2 wt.% silane concentration), CSFCM-2 (4 wt.% silane concentration), CSFCM-3 (6 wt.% silane concentration), CSFCM-4 (8 wt.% silane concentration), and CSFCM-5 (10 wt.% silane concentration).

### 2.2. Characterization of CSFCM-Reinforced Polymer Composites

#### 2.2.1. Density Measurement

Density was determined based on the Archimedes principle, utilizing a high-precision analytical balance (MP-5002, Shanghai Fangrui Instruments, Shanghai, China; accuracy ± 0.01 mg) equipped with a density determination kit. The experimental protocol was executed in a temperature-controlled chamber (25 ± 0.2 °C) using HPLC-grade water (resistivity > 18.2 MΩ·cm, TOC < 5 ppb) as the immersion medium. For each composite variant, five sequential measurements were conducted at 60 s intervals to ensure measurement stability. The composite density (*ρ_s_*) was determined according to the following equation:

(1) $$\rho_{s}=\frac{W_{a}}{W_{a}-W_{0}}\times \rho_{0}$$

where *W_a_* represents the sample mass in air (g), *W*_0_ represents the sample mass in water (g), and *ρ*_0_ denotes the density of water at the measurement temperature (g/cm^3^).

#### 2.2.2. Water Absorption Testing

The water absorption characteristics were investigated following the GB/T 24508-2009 standard [25]. The samples were first placed in a ventilated oven (LC-101-0B, Shanghai Lichen-BX Instrument Technology Co., Ltd., Shanghai, China)at 180 °C for 4 h with a heating rate of 5 °C/min. During the drying process, samples were positioned on aluminum foil with sufficient spacing (approximately 2 cm apart) to ensure uniform heat distribution and effective moisture removal. After the 4 h heat treatment, the oven was turned off and the samples were allowed to naturally cool to room temperature (23 ± 2 °C) inside the closed oven for approximately 6 h; to prevent thermal shock and moisture reabsorption, the conditioned samples were immersed in deionized water (resistivity > 18.2 MΩ·cm) at 23 ± 1 °C for 72 h to achieve saturation. After immersion, surface water was meticulously wiped using lint-free filter paper, and mass measurements were immediately performed using an analytical balance. The water absorption ratio (*W_t_*) was calculated according to the following equation [26]:

(2) $$W_{t}=\frac{M_{t}-M_{0}}{M_{0}}\times 100\%$$

where *M_t_* represents the saturated sample mass and *M*_0_ denotes the initial dry mass. Each measurement was performed in triplicate to ensure data reliability, and the mean values were reported with standard deviations.

#### 2.2.3. Apparent Porosity Determination

The composite samples were dried in a vacuum oven at 90 ± 1 °C until a constant mass was achieved. Subsequently, the samples were immersed in boiling distilled water (resistivity > 18.2 MΩ·cm) for 120 min. After cooling to 25 ± 0.5 °C in a thermostatic water bath, mass measurements were performed in both air and water using an analytical balance. The apparent porosity (*φ*) was calculated using the following equation [27]:

(3) $$\varphi =(\frac{G_{2}-G_{1}}{G_{2}-G_{3}})\times 100\%$$

where *G*_1_ represents the initial dry mass (g), *G*_2_ represents the saturation mass in air (g), and *G*_3_ represents the immersion mass in water (g). Eight parallel samples were tested for each composite formulation under controlled environmental conditions (temperature: 25 ± 1 °C; relative humidity: 50 ± 5%).

#### 2.2.4. Tribological Testing

All tribological tests were performed in a laboratory environment with controlled ambient conditions. The room temperature was maintained at 25 ± 2 °C using central air conditioning, and the relative humidity was monitored and maintained at 50 ± 5% using commercial dehumidifiers. These environmental parameters were recorded at the beginning and end of each test series to ensure consistent testing conditions. Prior to testing, all specimens were stored and conditioned in the same laboratory environment for at least 24 h to achieve equilibrium with the ambient conditions.

Friction and wear characteristics were determined using a JF151 constant-speed friction testing apparatus (Jilin Wangda Testing Equipment Co., Ltd., Changchun, China) following GB 5763-2018 standards [28]. The counterface friction discs were made from HT250-grade cast iron (with a predominantly pearlitic microstructure; hardness: HB180-220). Tests included thermal fade and recovery assessments across a temperature range of 100–350 °C and were performed at 0.98 MPa (normal pressure) and a rotational speed of 480 rpm, corresponding to a linear sliding velocity of 7.54 m/s at a mean friction radius of 150 mm. Fade resistance tests were conducted at six temperatures (100, 150, 200, 250, 300, and 350 °C). Recovery performance was assessed during controlled cooling from 300 °C to 100 °C at 50 °C intervals. The friction coefficient (*μ*) and specific wear rate (Δ*V*) were calculated using the following equations [29,30]:

(4) $$\mu =\frac{f}{P}$$

(5) $$\Delta V=\frac{1}{2\pi r}\times \frac{A}{R}\times \frac{d_{1}-d_{2}}{f}$$

where *f* represents the average friction force (N), *P* denotes the applied normal load (N), *r* is the effective friction radius (150 mm), *R* indicates the total revolution count (5000), *A* represents the nominal contact area (625 mm^2^), and *d*_1_ and *d*_2_ are the specimen thicknesses before and after testing (cm). To ensure statistical validity, each test condition was replicated three times.

#### 2.2.5. Thermal Stability Evaluation

The thermal stability characteristics of the composite samples were quantitatively evaluated through fade ratio (*F*) and recovery ratio (*R*) analyses based on the friction coefficient data obtained from high-temperature friction-wear tests. The fade resistance, representing the material’s ability to maintain stable friction performance under elevated temperatures, and recovery performance, indicating the material’s capability to restore its friction characteristics after thermal exposure, were calculated using the following equations [31]:

(6) $$F=\frac{\mu_{F100{}^{\circ}C}-\mu_{F350{}^{\circ}C}}{\mu_{F100{}^{\circ}C}}\times 100\%$$

(7) $$R=\frac{\mu_{R100{}^{\circ}C}}{\mu_{F100{}^{\circ}C}}\times 100\%$$

where *μ_F_*_100°C_ and *μ_F_*_350°C_ represent the friction coefficients measured at 100 °C and 350 °C during the fade test sequence, respectively, and *μ_R_*_100°C_ represents the friction coefficient measured at 100 °C during the recovery test phase. These parameters provide quantitative metrics for evaluating the thermal stability and reliability of the friction materials under dynamic temperature conditions, with lower fade ratios and higher recovery ratios indicating superior thermal stability performance.

#### 2.2.6. Morphological Observation

The surface morphology and microstructural characteristics of both the fiber materials and composites were examined using a field emission scanning electron microscope (SEM, EVO-18, Zeiss, Oberkochen, Germany). The observations were performed at an accelerating voltage of 15–20 kV and working distance of 8–12 mm. Prior to examination, all samples were sputter-coated with a thin gold layer (10–15 nm) to enhance surface conductivity. For fiber analysis, both untreated and silane-treated cotton stalk fibers were examined to evaluate the surface modification effects. The composite samples were analyzed to investigate the fiber–matrix interface and worn surface characteristics. Images were captured at magnifications ranging from 100× to 5000× to analyze the surface morphology of fibers, fiber–matrix interfaces, and wear tracks.

## 3. Results and Discussion

### 3.1. Density Characteristics

**Figure 3:**
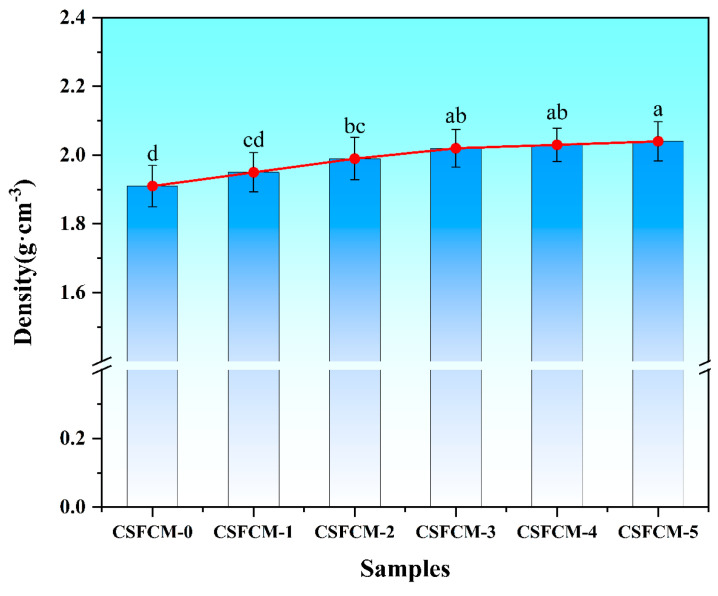
Density variation in composites with different silane treatment concentrations. Averages followed by different lowercase letters are significantly different according to LSD’s multiple range test at the significance level of 0.05. Error bars are standard deviation.

The density characteristics of CSFCM composites with varying silane treatment concentrations are presented in Figure 3. The density measurements reveal a moderate increasing trend from CSFCM-0 (1.92 g/cm^3^) to CSFCM-5 (2.08 g/cm^3^). Multiple comparison analysis using the LSD test indicates that CSFCM-5 exhibits significantly higher density than CSFCM-0 through CSFCM-2. CSFCM-3 and CSFCM-4 show intermediate density values with no significant difference between them, suggesting a transitional stage in the densification process.

This progressive density evolution can be attributed to the enhanced fiber–matrix interfacial bonding at higher silane concentrations, where more silane molecules form dense siloxane networks and promote better matrix penetration into the fiber surface. At lower silane concentrations (2–4 wt.%), the coupling agent initiates surface modification by replacing hydroxyl groups with organosilane molecules, showing no significant difference in density. The intermediate concentrations (6–8 wt.%) achieve optimal interface modification, where enhanced fiber–matrix adhesion facilitates efficient composite consolidation. However, at 10 wt.% concentration (CSFCM-5, ‘a’), excessive silane treatment may lead to the formation of densified siloxane networks, resulting in the highest observed density. Notably, due to the inherent porous cellular structure of bio-pre-treated corn stalk fibers, all CSFCM composites maintained relatively low densities (1.9–2.1 g/cm^3^), which are considerably lower than traditional friction materials (2.2–2.5 g/cm^3^). These density values align well with the findings reported by Liu et al. [24] and Nishino et al. [32] for natural fiber-based friction composites.

### 3.2. Water Absorption Characteristics

**Figure 4:**
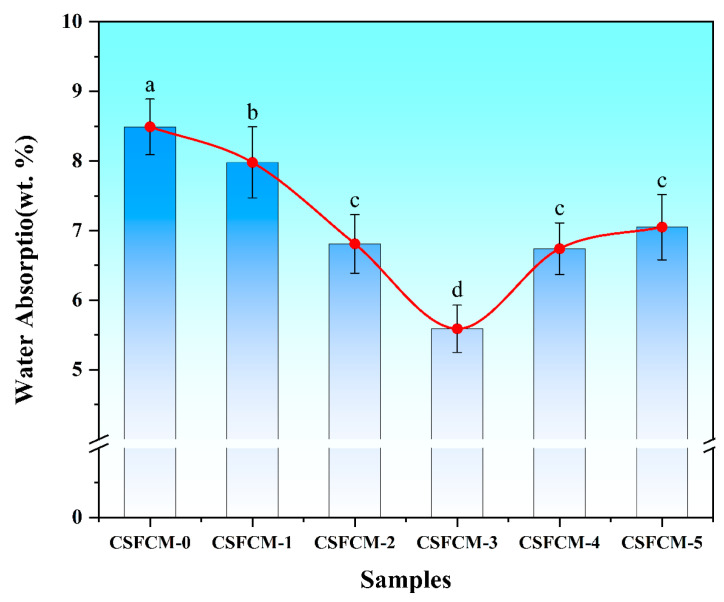
Effect of silane treatment concentration on the water absorption of CSFCM-reinforced composites. Averages followed by different lowercase letters are significantly different according to LSD’s multiple range test at the significance level of 0.05. Error bars are standard deviation.

The water absorption characteristics of CSFCM-reinforced composites with varying silane treatment concentrations are presented in Figure 4. The results show a U-shaped pattern, with water absorption values ranging from 8.49% for untreated specimens (CSFCM-0) to a minimum of 5.59% for specimens treated with 6 wt.% silane (CSFCM-3). At low silane concentrations (2–4 wt.%), the coupling agent modifies the fiber surface by replacing hydrophilic hydroxyl groups with hydrophobic organosilane units, as shown by the decrease in water absorption from 8.49% (CSFCM-0) to 7.98% (CSFCM-1) and 6.81% (CSFCM-2). The silane molecules undergo hydrolysis and bond with fiber surfaces, creating a hydrophobic layer that reduces water accessibility to the fiber structure [33].

At a moderate concentration (6 wt.%), an optimal surface coverage is achieved, with CSFCM-3 exhibiting the lowest water absorption of 5.59%, showing statistically significant decreases (*p* < 0.05) from CSFCM-0 through CSFCM-2, resulting in a 34.2% reduction compared to untreated specimens. This concentration provides sufficient silane molecules to form a complete siloxane layer through condensation of silane groups while maintaining uniform distribution. The silane treatment effectively reduces water absorption in polymer composite systems by eliminating surface hydroxyl groups on natural fibers and increasing the crystallinity of CSFCM [34]. At higher concentrations (8–10 wt.%), water absorption increases to 6.74% for CSFCM-4 and 7.05% for CSFCM-5, which were not significantly different from CSFCM-2 (*p* > 0.05). This increase is attributed to the formation of silane oligomers and irregular surface topology, which may contribute to increased water retention [35]. These results are consistent with the findings of Vilay et al. [36] in their study of bagasse fiber-reinforced composites.

All treated samples maintained lower water absorption than untreated CSFCM (8.49%), with 6 wt.% silane treatment providing optimal moisture resistance. This behavior reflects the effectiveness of surface modification in CSFCM-reinforced composites that have undergone biological pre-treatment.

### 3.3. Porosity Features

**Figure 5:**
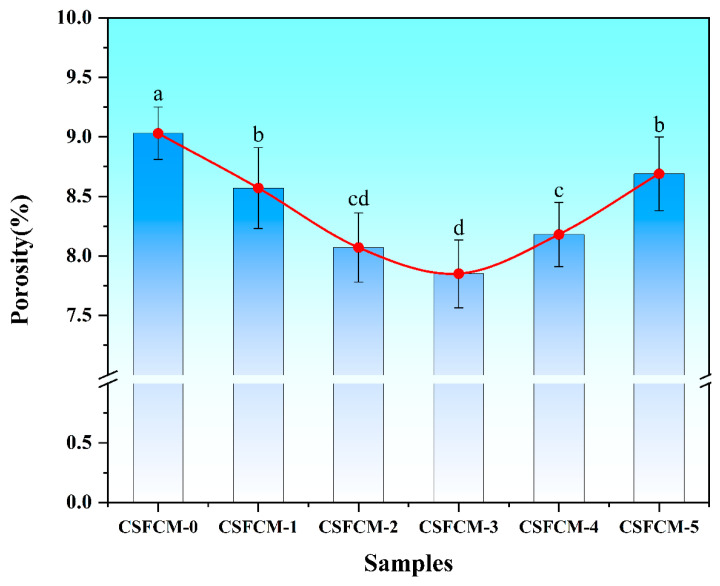
Apparent porosity of CSFCM composites as a function of silane treatment concentration. Averages followed by different lowercase letters are significantly different according to LSD’s multiple range test at the significance level of 0.05. Error bars are standard deviation.

Figure 5 presents the porosity characteristics of CSFCM composites with varying silane treatment concentrations. The apparent porosity demonstrated a relationship with water absorption behavior, reflecting the connection between microstructural features and macroscopic properties. The untreated specimen (CSFCM-0) exhibited a porosity of 9.03%, higher than conventional corn stalk fiber composites (6–8% before treatment). The porosity reached a minimum of 7.85% for the 6 wt.% silane treatment (CSFCM-3), and increased at higher treatment concentrations.

The elevated initial porosity of CSFCM compared to conventional corn stalk fibers stems from structural modifications induced by ruminant digestion. During ruminal fermentation, microbial systems selectively degrade hemicellulose and pectin components, leading to partial delignification and increased cellulose accessibility. This biological pre-treatment creates additional micropores and channels within the fiber structure. The enzymatic degradation of cell wall components results in a more open fiber architecture compared to untreated corn stalk fibers [10].

The porosity values follow a similar trend to water absorption, showing statistically significant decreases (*p* < 0.05) from CSFCM-0 (9.03%) through CSFCM-2. The notably higher initial porosity compared to conventional corn stalk fiber composites (6–8%) stems from structural modifications during ruminal fermentation, where microbial systems selectively degrade hemicellulose and pectin components, creating additional micropores and channels [37]. CSFCM-3 achieves the minimum porosity of 7.85%, demonstrating optimal interfacial bonding at 6 wt.% silane treatment. At this moderate concentration, silane treatment establishes effective chemical bonding at the interface, reducing interfacial voids while enhancing fiber surface hydrophobicity. However, CSFCM-4 and CSFCM-5 show progressive increases in porosity, with CSFCM-5 reaching 8.69% (statistically similar to CSFCM-1, *p* > 0.05). This increase at higher concentrations (8–10 wt.%) can be attributed to the formation of irregular interfacial structures and new defects. The observed reduction in porosity with silane treatment is consistent with the findings of Tonoli et al. [38], who also demonstrated decreased apparent porosity in silane-treated cellulose pulp fiber composites, confirming the effectiveness of silane treatment in reducing composite porosity.

The 6 wt.% silane treatment (CSFCM-3) optimized the interfacial structure and porosity characteristics, though the absolute porosity values reflect the structural features inherited from biological pre-treatment. These findings indicate that CSFCM-based composites maintain distinct microstructural characteristics despite surface modification.

### 3.4. Tribological Performance

**Figure 6:**
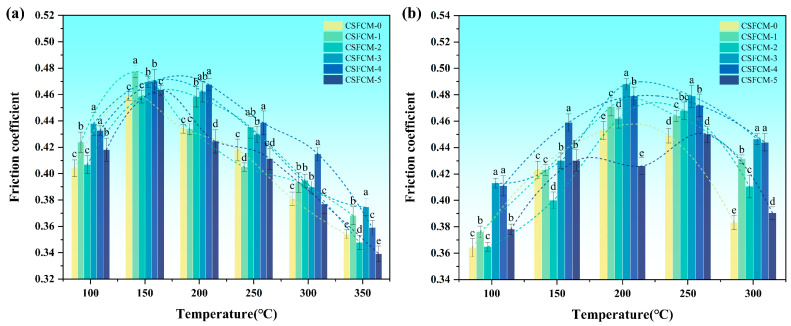
Friction coefficient of CSFCM composites in the fade test (**a**) and recovery test (**b**). Averages followed by different lowercase letters are significantly different according to LSD’s multiple range test at the significance level of 0.05. Error bars are standard deviation.

The friction coefficients of CSFCM-reinforced composites varied significantly with temperature and silane treatment concentrations (Figure 6a). Statistical analysis reveals that CSFCM-3 and CSFCM-4 (6–8 wt.% silane) consistently demonstrate significant improvements (*p* < 0.05) in friction stability across all temperature ranges, while other treatments show significant but irregular variations from the control. In the temperature range of 100–150 °C, all specimens exhibited increasing friction coefficients due to the initial accumulation of wear debris and formation of primary friction films. The softening of the phenolic resin matrix enhanced the contact area with the counterpart, facilitating wear particle embedding and friction film formation.

Above 150 °C, a notable decrease in friction coefficients was observed across all specimens. This reduction stemmed from the thermal decomposition of the phenolic resin matrix and the carbonization of residual organic matter in biologically treated CSFCM. The carbonization process generated lubricating carbonaceous layers at the friction interface, with the effect intensifying between 250 and 350 °C [39]. The extensive development of stable secondary plateaus indicated efficient tribomechanical mixing and debris consolidation under dynamic loading conditions. Below 250 °C, the wear mechanism was primarily dominated by mechanical interactions, characterized by limited surface degradation and effective stress distribution through the modified interface. As temperatures increased above 250 °C, the previously described carbonization process of CSFCM generated complex tribological effects: while the carbonaceous layers provided some lubrication, they also modified the interface structure and wear mechanisms. These carbonized layers underwent continuous formation and destruction cycles, creating a dynamic tribological system where wear debris became incorporated into transfer films through tribomechanical mixing. The specimen thus demonstrated a transition to mild oxidative wear, characterized by the formation of protective tribologically modified surface layers, though with reduced stability at higher temperatures. This temperature-dependent behavior explains the observed variations in wear rates and supports the material’s optimal application in low-to-medium temperature environments where mechanical wear mechanisms predominate.

Specimens treated with 4–8 wt.% silane (CSFCM-2, CSFCM-3, and CSFCM-4) exhibited superior friction stability, particularly at 200 °C. The appropriate silane treatment enhanced fiber–matrix interfacial bonding and regulated CSFCM carbonization behavior. The chemical bond network formed by silane coupling agents moderated the carbonization process, resulting in uniform carbonaceous lubricating layers and stable friction films [40]. Insufficient silane treatment (CSFCM-0 and CSFCM-1) led to weak interfacial bonding and premature interface failure at elevated temperatures. Conversely, excessive silane treatment (CSFCM-5) created discontinuous interfacial structures through molecular self-polymerization, leading to stress concentration points and unstable friction performance at high temperatures.

The recovery characteristics during cooling (350 °C to 100 °C) revealed distinct behavior patterns (Figure 6b). Initial cooling (300–250 °C) induced significant increases in friction coefficients due to the solidification of decomposed resin products and formation of stable carbonaceous structures. The intermediate range (250–200 °C) exhibited relatively stable friction coefficients, indicating equilibrium between friction film consolidation and structural reorganization. Further cooling (200–100 °C) resulted in declining friction coefficients, attributed to increased friction film brittleness and thermal contraction effects. Similar cooling-induced friction behavior patterns have been reported in other plant fiber-reinforced friction materials, where Elen et al. [41] observed comparable friction coefficient variations in hemp fiber composites, and Mylsamy et al. [42] documented analogous thermal recovery characteristics in Coccinia Indica fiber-based systems, confirming these phenomena as characteristic features of plant fiber friction materials. CSFCM-3 and CSFCM-4 (6–8 wt.% silane) demonstrated optimal recovery performance throughout the cooling cycle, reflecting enhanced chemical bonding and a uniform stress distribution. Lower silane concentrations (0–4 wt.%) showed limited improvement in friction film stability during cooling, confirming the importance of optimal silane treatment for enhanced thermal recovery characteristics [43].

The friction coefficient trends reveal three distinct temperature-dependent regions: (1) an initial increase from 100 to 150 °C due to wear debris accumulation and friction film formation, (2) a decline between 150 and 250 °C attributed to matrix softening and carbonization, and (3) relative stabilization above 250 °C. CSFCM-3 maintains the most stable friction coefficients (0.35–0.45) across all temperature ranges, indicating superior thermal stability and consistent tribological performance.

### 3.5. Thermal Stability of Friction Properties

**Figure 7:**
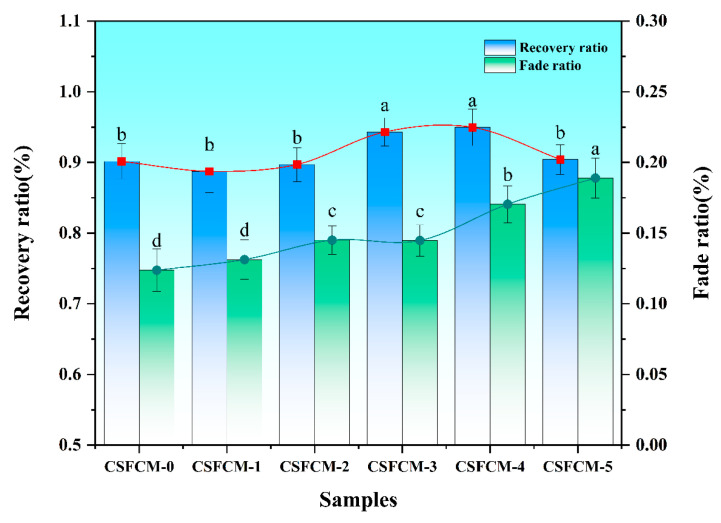
Fade ratio and recovery ratio characteristics of CSFCM-reinforced composites with varying silane treatment concentrations. Averages followed by different lowercase letters are significantly different according to LSD’s multiple range test at the significance level of 0.05. Error bars are standard deviation.

The fade and recovery characteristics of CSFCM-reinforced composites were systematically evaluated to assess their thermal stability and friction performance reliability. Figure 7 presents the fade ratio and recovery ratio variations across different silane treatment concentrations. The results revealed distinct trends in both parameters, reflecting the complex interplay between surface modification and thermal response mechanisms.

The fade ratio demonstrated an overall increasing trend from CSFCM-0 (12.38%) to CSFCM-5 (18.90%), with intermediate treatments showing moderate values: CSFCM-1 (13.13%), CSFCM-2 (14.51%), CSFCM-3 (14.48%), and CSFCM-4 (17.04%). Statistical analysis using LSD’s multiple range test (*p* < 0.05) revealed distinct groupings in fade ratio performance. CSFCM-5 exhibited a significantly higher fade ratio compared to all other treatments. CSFCM-4 showed a significantly lower fade ratio than CSFCM-5 but remained significantly higher than other treatments. CSFCM-2 and CSFCM-3 demonstrated statistically similar intermediate fade ratios, while CSFCM-0 and CSFCM-1 showed no significant difference from each other but were significantly lower than all other treatments. The lower fade ratio in untreated specimens (CSFCM-0) can be attributed to the preservation of natural hydroxyl groups on fiber surfaces, which facilitate the formation of hydrogen-bonded networks during thermal exposure.

The recovery ratio analysis revealed superior performance in moderately to highly treated specimens, with CSFCM-4 achieving the highest recovery ratio of 94.97%, followed closely by CSFCM-3 at 94.31%. Statistical analysis using LSD’s multiple range test (*p* < 0.05) showed that CSFCM-3 and CSFCM-4 exhibited significantly higher recovery ratios compared to other treatments. In contrast, untreated and low-concentration specimens showed lower recovery values: CSFCM-0 (90.16%), CSFCM-1 (88.74%), and CSFCM-2 (89.71%), with no significant differences among them. This enhancement in recovery characteristics can be attributed to the formation of thermally stable siloxane networks at the fiber–matrix interface. These networks, when properly developed through optimal silane treatment, provide resilient anchor points that facilitate the reformation of friction films during cooling. Despite showing a relatively high fade ratio, CSFCM-5 maintained a reasonable recovery ratio of 90.43%.

The moderate treatment range (CSFCM-3 and CSFCM-4) achieved an optimal balance between fade resistance and recovery performance, indicating that these concentrations create interfacial structures capable of maintaining both thermal stability and reversible friction characteristics. This balanced performance likely results from the formation of optimally spaced siloxane networks that provide sufficient thermal stability while maintaining necessary molecular mobility for effective friction film regeneration during cooling cycles.

These findings suggest that while increased silane treatment may slightly compromise fade resistance, it significantly enhances the material’s ability to recover its friction characteristics after thermal exposure. The combination of moderate fade ratios (12.38–18.90%) and excellent recovery performance (up to 94.97% for optimally treated specimens) particularly supports the application of these materials in low-to-medium-temperature operating conditions. Under such conditions, where thermal loads are less severe, the superior recovery characteristics and controlled fade behavior would contribute to stable and reliable friction performance. This makes CSFCM-reinforced composites especially suitable for light-duty applications such as agricultural machinery, light commercial vehicles, or other systems operating under moderate thermal loads. Therefore, the balanced performance achieved through optimal silane treatment (CSFCM-3 and CSFCM-4) represents a promising direction for developing sustainable friction materials specifically tailored for low-to-medium-temperature applications.

The fade ratio shows a gradual increase with silane concentration from 12.38% (CSFCM-0) to 18.90% (CSFCM-5), while the recovery ratio peaks at 94.97% for CSFCM-4. This inverse relationship between fade and recovery performance suggests that moderate silane treatment (6–8 wt.%) achieves optimal balance between thermal stability and friction recovery capabilities. Compared to previous studies on corn straw fiber composites by Liu et al. [24] and agave fiber-reinforced materials by Wu et al. [44], the present composites demonstrated superior fade and recovery characteristics, demonstrating the positive effects of biological pre-treatment on corn straw fiber performance in friction applications.

### 3.6. Wear Rate Behavior

**Figure 8:**
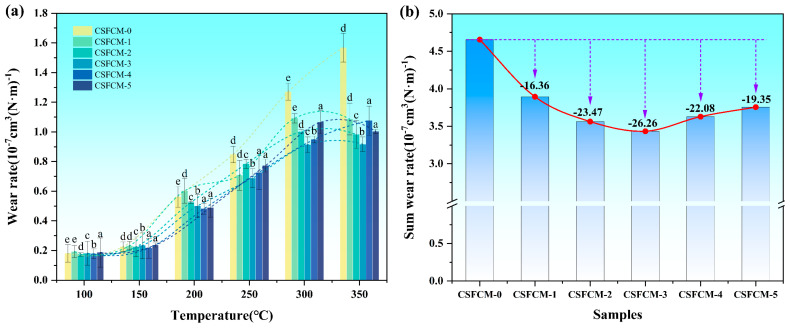
Wear behavior of CSFCM composites: (**a**) variation in wear rates with temperature for different silane concentrations (0–10 wt.%); (**b**) correlation between total wear rate and silane treatment concentration. Averages followed by different lowercase letters are significantly different according to LSD’s multiple range test at the significance level of 0.05. Error bars are standard deviation.

The wear resistance of CSFCM-reinforced composites was systematically evaluated across a temperature range of 100–350 °C, as presented in Figure 8. The results revealed complex temperature-dependent wear behaviors that varied significantly with silane treatment concentration. The wear behavior exhibited three distinct temperature-dependent stages. At low temperatures (100–150 °C), all specimens demonstrated relatively low and stable wear rates ranging from 0.17 to 0.24 × 10^−7^ cm^3^ (N·m)^−1^. Statistical analysis using LSD’s multiple range test (*p* < 0.05) showed no significant differences among different treatments in this temperature range, suggesting that under mild thermal conditions, the wear mechanism was primarily dominated by mechanical interactions rather than interfacial properties. A critical transition occurred in the intermediate temperature range (200–250 °C), where wear rates increased dramatically. For instance, CSFCM-0 showed a wear rate increase from 0.226 × 10^−7^ cm^3^ (N·m)^−1^ at 150 °C to 0.562 × 10^−7^ cm^3^ (N·m)^−1^ at 200 °C, representing a 149% increase. This transition likely corresponds to the thermal softening point of the phenolic matrix and the onset of interface degradation.

In the high-temperature region (300–350 °C), statistical analysis revealed significant differences in wear behavior among treatments. CSFCM-2, CSFCM-3, CSFCM-4, and CSFCM-5 showed significantly lower wear rates compared to untreated specimens (*p* < 0.05), while no significant differences were observed between CSFCM-0 and CSFCM-1. The untreated CSFCM-0 exhibited the most severe wear rate increase, rising from 0.181 to 1.568 × 10^−7^ cm^3^/(N·m)^−1^, indicating the poor thermal stability of the untreated interface. In contrast, CSFCM-3 demonstrated the most stable wear progression, while CSFCM-2 exhibited exceptional high-temperature stability with the lowest wear rate at 350 °C (0.853 × 10^−7^ cm^3^(N·m)^−1^). CSFCM-4 and CSFCM-5 showed moderate improvements over CSFCM-0 but could not match the performance of CSFCM-2 and CSFCM-3, suggesting that excessive silane treatment may compromise wear resistance.

The wear rate trends exhibit distinct temperature-dependent behaviors (Figure 8a). At low temperatures (100–150 °C), all specimens show relatively stable and low wear rates (0.17–0.24 × 10^−7^ cm^3^(N·m)^−1^). A critical transition occurs between 200 and 250 °C, marked by sharp increases in wear rates, particularly evident in untreated specimens. CSFCM-3 demonstrates the most stable wear progression across all temperature ranges, while CSFCM-0 shows the most severe wear rate increase at elevated temperatures.

The correlation between total wear rate and silane treatment concentration (Figure 8b) reveals an optimal point at 6 wt.% (CSFCM-3), achieving the lowest total wear rate of 3.433 × 10^−7^ cm^3^ (N·m)^−1^, representing a 26.26% reduction compared to untreated specimens. This optimum suggests that moderate silane treatment creates ideal interface conditions for wear resistance, while both insufficient (<4 wt.%) and excessive (>8 wt.%) treatment result in suboptimal wear performance improvements of only 16.36% and 19.36%, respectively.

Analysis of the total wear rate revealed that CSFCM-3 achieved the lowest total wear rate (3.433 × 10^−7^ cm^3^ (N·m)^−1^), representing a 26.26% reduction compared to CSFCM-0, followed by CSFCM-2 and CSFCM-4. This trend indicates that moderate silane concentrations (4–6 wt.%) provide optimal interface modification for enhanced wear resistance. The improvement in wear resistance showed a non-linear relationship with silane concentration, where both insufficient (CSFCM-1) and excessive (CSFCM-5) treatment resulted in suboptimal performance improvements of 16.36% and 19.36%, respectively.

These wear characteristics suggest that silane treatment at an appropriate concentration (particularly CSFCM-3) creates an interface structure capable of maintaining mechanical integrity under elevated temperatures while providing effective stress distribution during the wear process. The superior wear resistance, especially at higher temperatures, further supports the application potential of these materials in moderate-temperature friction applications where controlled wear behavior is crucial for long-term performance reliability.

### 3.7. Fiber Surface Morphology Analysis

**Figure 9:**
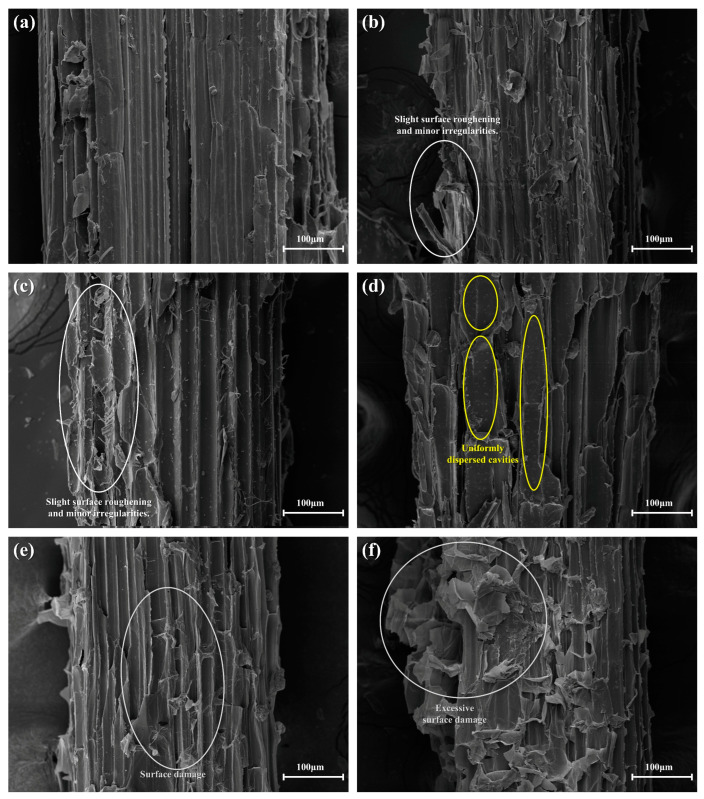
SEM micrographs showing surface morphology of CSFCM fibers treated with different silane concentrations: (**a**) untreated CSFCM; (**b**) 2 wt.% silane-treated CSFCM; (**c**) 4 wt.% silane-treated CSFCM; (**d**) 6 wt.% silane-treated CSFCM; (**e**) 8 wt.% silane-treated CSFCM; (**f**) 10 wt.% silane-treated CSFCM. All images were captured at 200× magnification.

Scanning electron microscopy (SEM) analysis revealed progressive changes in CSFCM surface morphology across different silane treatment concentrations (Figure 9). The untreated CSFCM (Figure 9a) displayed a relatively smooth and regular surface texture, characteristic of fibers that have undergone biological pre-treatment through ruminant digestion.

At low silane concentrations (2–4 wt.%, Figure 9b,c), the fiber surfaces began exhibiting initial signs of modification, characterized by slight surface roughening and minor irregularities. These early morphological changes suggest that the chemical treatment started to affect the fiber surface structure, though the modifications remained relatively modest. This initial surface modification corresponds to the moderate improvements in physical properties, as evidenced by the slight reductions in water absorption from 8.49% to 7.98% and apparent porosity from 9.03% to 8.65%. These surface modification patterns differ from those observed by Liu et al. [24] for conventional silane-treated corn stalk fibers, which typically show more aggressive surface etching even at lower treatment concentrations. The moderate surface modifications observed in CSFCM can be attributed to the protective effects of biological pre-treatment, which preserves the fundamental fiber structure while allowing controlled chemical modification.

The optimal treatment concentration (6 wt.%, Figure 9d) produced the most uniform surface modification pattern. The fiber surfaces displayed consistent roughness with evenly distributed microscale texturing and small, uniformly dispersed cavities. This regular surface modification pattern directly correlates with the superior performance characteristics observed, including the 34.2% reduction in water absorption and decreased apparent porosity to 7.85%. The uniform surface modification also contributes to the enhanced tribological properties, as demonstrated by the 26.26% reduction in total wear rate and improved thermal stability with a 94.31% recovery ratio.

At higher silane concentrations, particularly 8–10 wt.% (Figure 9e,f), more aggressive surface modification became evident. The 8 wt.% treatment (Figure 9e) resulted in increased surface roughness while maintaining relative uniformity across the fiber surface. However, at 10 wt.% concentration (Figure 9f), excessive surface damage was observed, manifesting as severe irregularities and significant structural deterioration of the fiber surface. This surface degradation explains the increased water absorption (7.05%) and apparent porosity (8.69%) at higher treatment concentrations, as the irregular surface features and structural defects create additional pathways for water penetration and void formation.

The surface morphology analysis provides critical insights into the structure–property relationships observed in CSFCM-reinforced composites. The optimal surface modification achieved at 6 wt.% treatment represents a crucial balance between enhanced surface roughness for mechanical interlocking and preserved fiber structural integrity. At excessive treatment concentrations (8–10 wt.%), the severe surface irregularities and structural deterioration create unfavorable conditions for the formation of uniform silane coupling layers on the fiber surface. These irregular surface features prevent the even distribution and effective bonding of silane molecules, explaining the reduction in physical and mechanical properties at higher concentrations. This correlation between surface modification and treatment concentration provides a fundamental understanding of the observed performance trends and validates the selection of 6 wt.% as the optimal treatment concentration for CSFCM-reinforced friction materials.

### 3.8. Surface Morphology and Wear Mechanisms

**Figure 10:**
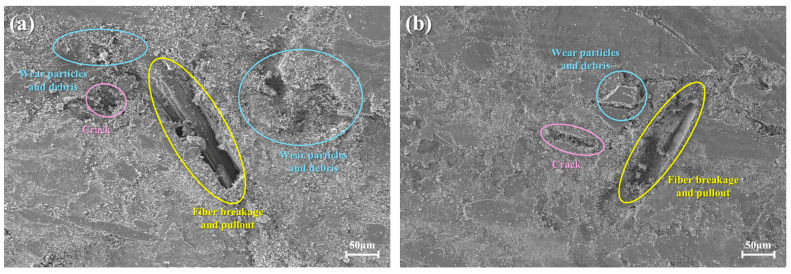

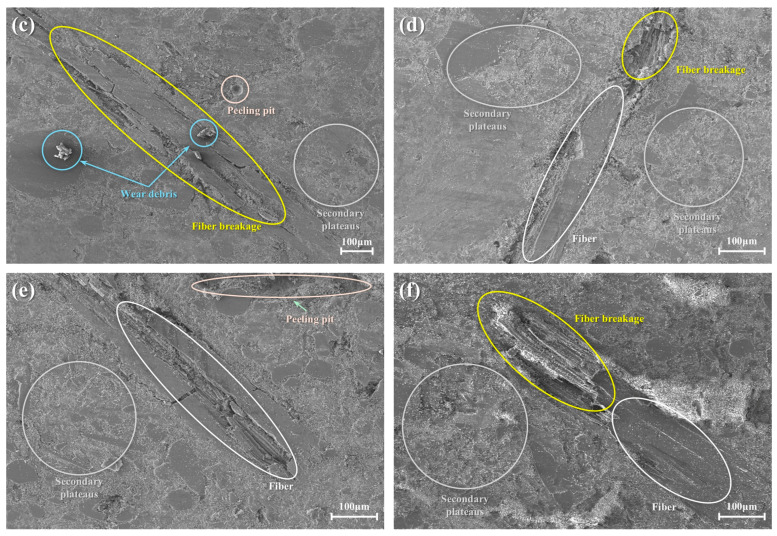
Surface morphology of CSFCM-reinforced composites: (**a**) CSFCM-0; (**b**) CSFCM-1; (**c**) CSFCM-2; (**d**) CSFCM-3; (**e**) CSFCM-4; (**f**) CSFCM-5.

The tribological behavior and wear mechanism evolution of CSFCM-reinforced composites were characterized through high-resolution scanning electron microscopy (SEM) analysis (Figure 10). The investigation revealed distinct wear mechanism transitions across varying silane treatment concentrations, encompassing adhesive, abrasive, fatigue, and delamination wear modes.

Microstructural analysis of untreated specimens (CSFCM-0, Figure 10a) revealed predominant adhesive wear characteristics, manifested through extensive interfacial delamination and fiber extraction. The inadequate fiber–matrix interfacial strength resulted in interfacial debonding under tribological loading, generating stress concentration sites that initiated microcrack formation. The liberated fiber fragments functioned as third-body abrasive elements, facilitating a transition from adhesive to three-body abrasive wear mechanisms, evidenced by bimodal wear debris distribution comprising fine adhesive particles and coarse fiber fragments.

CSFCM-1 specimens (Figure 10b) exhibited transitional wear behavior characterized by modified wear particle morphology despite persistent fiber detachment. The observed wear particle size distribution shift and reduced fine debris generation indicated a mechanistic transition from adhesive-dominated to abrasive-dominated wear. However, persistent microcrack propagation indicated suboptimal interface modification for fatigue resistance. This wear mechanism evolution differs from that reported by Ma et al. [29] for conventional natural fiber composites, where abrasive wear typically dominates throughout the wear process. The unique transition to controlled delamination wear in CSFCM composites can be attributed to the enhanced fiber–matrix interface achieved through the combination of biological pre-treatment and optimized silane modification.

A fundamental mechanistic transformation was observed in CSFCM-2 (Figure 10c), characterized by the emergence of delamination wear as the primary degradation mode. The initiation of secondary plateau formation indicated a transition towards a stabilized tribological regime, where compacted wear debris contributed to load distribution. The presence of characteristic delamination cavities suggests subsurface crack nucleation and propagation that is consistent with delamination wear theory, facilitating controlled material removal processes.

CSFCM-3 (Figure 10d) exhibited optimized tribological behavior through synergistic wear mechanism integration. The extensive development of stable secondary plateaus indicated efficient tribomechanical mixing and debris consolidation under dynamic loading conditions. Limited surface degradation under sustained loading suggested effective stress distribution through the modified interface. The specimen demonstrated a transition to mild oxidative wear, characterized by the formation of protective tribologically modified surface layers.

Microstructural analysis of CSFCM-4 (Figure 10e) revealed enhanced subsurface fatigue mechanisms despite maintaining fiber–matrix adhesion, manifested through increased delamination cavity dimensions. This observation suggests that excessive interfacial rigidity may compromise cyclic strain accommodation capacity, accelerating subsurface crack propagation. CSFCM-5 (Figure 10f) further exemplified this trend, where despite achieving optimal fiber–matrix bonding, enhanced surface deterioration and secondary plateau irregularity indicated severe wear regime reversion, attributed to interface embrittlement.

The systematic wear mechanism evolution across silane treatment concentrations elucidates the critical influence of interfacial properties on tribological behavior. The transition sequence from severe adhesive and three-body abrasive wear in untreated specimens to controlled delamination and oxidative wear in optimally treated samples, followed by enhanced fatigue wear at elevated treatment concentrations, demonstrates the complex interdependence between interface modification parameters and wear mechanisms.

Quantitative image analysis of SEM micrographs was performed using ImageJ software (Version 1.54g) to characterize key microstructural features. For each specimen type, measurements were taken from at least 20 different regions across multiple samples. In untreated specimens (CSFCM-0), wear debris exhibited diverse morphological characteristics: large debris fragments averaged 82 ± 18 μm, while fine wear particles showed dimensions of 7.2 ± 2.8 μm. Some exceptional large debris fragments reached 145 ± 12 μm, particularly in regions experiencing severe delamination. Surface examination revealed delamination pits with diameters varying 55 ± 12 μm, and surface cracks extending from 165 ± 35 μm to 242 ± 28 μm in length. These cracks predominantly appeared along the sliding direction, often interconnecting with delamination regions. Under optimal treatment conditions (CSFCM-3), the wear mechanism demonstrated a notable shift in debris characteristics. The proportion of large debris (>50 μm) decreased significantly, while fine wear particles (6.8 ± 2.2 μm) became more prevalent. The delamination pits were less frequent and showed reduced dimensions of 42 ± 8 μm. Surface cracks were also less extensive, typically ranging from 125 ± 28 μm to 185 ± 32 μm in length. Specimens with excessive treatment (CSFCM-5) showed increased formation of large wear debris (95 ± 22 μm) and more pronounced surface damage, with delamination pits expanding to 68 ± 15 μm and crack lengths extending up to 262 ± 42 μm.

The microscopic results support a comprehensive tribological model in which optimized silane treatment (CSFCM-3) establishes interfacial characteristics that facilitate multiple synergistic wear mechanisms. The controlled delamination wear proceeds through stable subsurface crack propagation pathways, while efficient tribomechanical mixing and wear debris consolidation generate load-bearing secondary plateaus. The concurrent formation of protective tribofilms through mild oxidative wear mechanisms further enhances surface protection. This optimized integration of wear mechanisms, achieved through precise interface modification, leads to a superior tribological performance compared to specimens with insufficient treatment (characterized by predominant adhesive wear) or excessive treatment (dominated by accelerated fatigue wear mechanisms). The established relationship between silane treatment concentration and wear mechanism evolution provides essential information for developing high-performance friction materials with controlled interface properties.

## 4. Conclusions

This investigation systematically examined the effects of silane treatment at varied concentrations (2–10 wt.%) on corn stalk fibers extracted from cattle manure (CSFCM) for the development of friction materials. Comprehensive analysis of physicochemical, tribological, and microstructural characteristics yielded the following significant findings:

(1) The best interface modification was achieved at a 6 wt.% silane concentration (CSFCM-3), resulting in a substantial enhancement of the physical properties. This optimization manifested in a 34.2% reduction in water absorption capacity and a decrease in the apparent porosity from 9.03% to 7.85%. The modified composites maintained a favorable density range of 1.9–2.1 g/cm^3^, significantly below that of conventional friction materials (2.2–2.5 g/cm^3^).
(2) The tribological characterization of CSFCM-3 demonstrated exceptional performance stability, as the friction coefficients were maintained within 0.35–0.45 across the temperature range of 100–350 °C. Thermal stability analysis revealed superior performance parameters, including a fade ratio of 14.48% and a recovery ratio of 95%. The total wear rate exhibited a substantial reduction of 26.26%, reaching 3.433 × 10^−7^ cm^3^ (N·m)^−1^ compared to unmodified specimens, indicating enhanced wear resistance under dynamic thermal conditions.
(3) Microstructural analysis highlighted that there were fundamental transformations in wear mechanisms following optimal silane modification. The treatment facilitated the formation of uniformly distributed, densified secondary plateaus with enhanced matrix adhesion, contributing to improved tribological stability. A significant transition from severe adhesive and three-body abrasive wear to controlled two-body abrasive wear was observed, characterized by reduced delamination cavity formation, uniformly distributed shallow plowing patterns, and refined wear debris morphology conducive to stable tribofilm development.
(4) The integration of biological pre-treatment through ruminant digestion with optimized silane modification establishes an innovative protocol for developing high-performance friction materials from agricultural waste streams. The enhanced cellulose content and modified fiber architecture resulting from biological pre-treatment, combined with precisely controlled chemical modification, creates a novel platform for sustainable material development. This investigation provides valuable technical insights for industrial applications of environmentally friendly composites, particularly in validating the feasibility of cattle manure-derived corn stalk fibers for friction materials. The demonstrated performance improvements and environmental benefits make this technology particularly attractive for sustainable friction material manufacturing, though challenges remain in scaling up production and managing economic impacts. This research involves the coordination between agricultural and industrial sectors, and faces challenges in optimizing processing parameters for different plant fiber composites. Beyond tribological applications, further research is needed to explore its potential in lightweight, high-strength, and eco-friendly composite materials. Key aspects requiring investigation include optimization of the fiber–matrix interface through surface modification techniques, enhancement of mechanical properties through fiber orientation control and distribution, and development of standardized processing protocols for various agricultural waste streams.

The tribological and mechanical properties obtained in this study meet the national standards for brake friction materials, demonstrating the feasibility of these composites as brake materials. However, considering their high-temperature performance characteristics, these materials are more suitable for braking environments with moderate loads, specifically in light-load or medium-low temperature conditions. Such applications could include light vehicles, agricultural machinery, or industrial conveying equipment where severe thermal conditions are not typically encountered. Moreover, this study provides valuable insights for expanding the application potential of cattle manure straw fiber composites in other working conditions, particularly where moderate mechanical and tribological properties are required.

This investigation demonstrates the efficacy of integrating biological pre-treatment with chemical modification strategies while establishing new methodologies for developing sustainable, high-performance friction materials from agricultural waste resources. The combination of environmental sustainability and enhanced performance characteristics provides a foundation for future developments in eco-conscious friction material design.

## Figures and Tables

**Table 1:** Material composition and weight percentages of the CSFCM-based composite.

Component Type	Component	Weight Percentage
Binder	Phenolic resin	14 ± 0.5 wt.%
Modifier	Graphite	8 ± 0.3 wt.%
	Antimony sulfide	3 ± 0.2 wt.%
	Bauxite	7 ± 0.3 wt.%
	Zinc stearate	2 ± 0.1 wt.%
	Porous iron powder	10 ± 0.4 wt.%
	Petroleum coke	5 ± 0.2 wt.%
Fibrous reinforcement	Compound mineral fibers	19 ± 0.5 wt.%
	CSFCM	6 ± 0.2 wt.%
Filler	Vermiculite powder	5 ± 0.2 wt.%
	Friction powder	2 ± 0.1 wt.%
	Barium sulfate	19 ± 0.5 wt.%

## Data Availability

The original contributions presented in this study are included in the article. Further inquiries can be directed to the corresponding authors.

## References

[1] Singh N., Walker T.R. (2024). Plastic recycling: A panacea or environmental pollution problem. NPJ Mater. Sustain..

[2] Dalmis R. (2023). Description of a new cellulosic natural fiber extracted from *Helianthus tuberosus* L. as a composite reinforcement material. Physiol. Plant..

[3] Bhat S.I., Mobin M., Islam S., Zehra S. (2024). Recent advances in anticorrosive coatings based on sustainable polymers: Challenges and perspectives. Surf. Coat. Technol..

[4] Zwawi M. (2021). A review on natural fiber bio-composites, surface modifications and applications. Molecules.

[5] Kamarudin S.H., Mohd Basri M.S., Rayung M., Abu F., Ahmad S.B., Norizan M.N., Osman S., Sarifuddin N., Desa M.S.Z.M., Abdullah L.C. (2022). A review on natural fiber reinforced polymer composites (NFRPC) for sustainable industrial applications. Polymers.

[6] Akter M., Uddin M.H., Tania I.S. (2022). Biocomposites based on natural fibers and polymers: A review on properties and potential applications. J. Reinf. Plast. Compos..

[7] Smerald A., Rahimi J., Scheer C. (2023). A global dataset for the production and usage of cereal residues in the period 1997–2021. Sci. Data.

[8] Font-Palma C. (2019). Methods for the Treatment of Cattle Manure—A Review. C.

[9] Petzel E.A., Titgemeyer E.C., Smart A.J., Hales K.E., Foote A.P., Acharya S., Bailey E.A., Held J.E., Brake D.W. (2019). What is the digestibility and caloric value of different botanical parts in corn residue to cattle?. J. Anim. Sci..

[10] Xu Q., Zhong H., Zhou J., Wu Y., Ma Z., Yang L., Wang Z., Ling C., Li X. (2021). Lignin degradation by water buffalo. Trop. Anim. Health Prod..

[11] Li K., Yang Z., Zhang Y., Li Y., Lu L., Niu D. (2022). Effect of pretreated cow dung fiber on mechanical and shrinkage properties of cementitious composites. J. Clean. Prod..

[12] Yang Z., Li K., Yan X., Wu W., Briseghella B., Marano G.C. (2024). Characterization and value-added applications of natural cellulose fibers derived from cow dung in cementitious composites. Cellulose.

[13] Yang X., Li L., Zhao W., Wang M., Yang W., Tian Y., Zheng R., Deng S., Mu Y., Zhu X. (2023). Characteristics and functional application of cellulose fibers extracted from cow dung wastes. Materials.

[14] Ma Y., Wu S., Zhuang J., Tong J., Qi H. (2019). Tribological and physio-mechanical characterization of cow dung fibers reinforced friction composites: An effective utilization of cow dung waste. Tribol. Int..

[15] Wu S., Guo M., Zhao J., Wu Q., Zhuang J., Jiang X. (2022). Characterization of the mechanical and morphological properties of cow dung fiber-reinforced polymer composites: A comparative study with corn stalk fiber composites and sisal fiber composites. Polymers.

[16] Reddy T.R.K., Rao T.S., Suvarna R.P. (2024). Studies on thermal characteristics of cow dung powder filled glass-polyester hybrid composites. Compos. Part B.

[17] Yang X., Li L., Zhao W., Tian Y., Zheng R., Deng S., Mu Y. (2023). The influence of potassium hydroxide concentration and temperature on pulp characteristics and cow dung-based paper performance. J. Nat. Fibers.

[18] Li K., Yang Z., Yan X., Xu L., Briseghella B., Marano G.C. (2023). Effect of Modified Cow Dung Fibers on Strength and Autogenous Shrinkage of Alkali-Activated Slag Mortar. Materials.

[19] Fasake V., Dashora K. (2021). A sustainable potential source of ruminant animal waste material (dung fiber) for various industrial applications: A review. Bioresour. Technol. Rep..

[20] Fasake V., Dashora K. (2024). Characterization of raw and anaerobic digested cattle dung fibers: A sustainable source of non-wood material. Biomass Convers. Biorefin..

[21] Kudva A., Mahesha G.T., Hegde S., Pai D. (2024). Influence of chemical treatment of Bamboo fibers on the vibration and acoustic characterization of Carbon/Bamboo fiber reinforced hybrid composites. Mater. Res. Express.

[22] El Boustani M., Lebrun G., Brouillette F., Belfkira A. (2017). Effect of a solvent-free acetylation treatment on reinforcements permeability and tensile behaviour of flax/epoxy and flax/wood fibre/epoxy composites. Can. J. Chem. Eng..

[23] Zhang C., Liu L., Zeng G., Huang D., Lai C., Huang C., Wei Z., Li N., Xu P., Cheng M. (2014). Utilization of nano-gold tracing technique: Study the adsorption and transmission of laccase in mediator-involved enzymatic degradation of lignin during solid-state fermentation. Biochem. Eng. J..

[24] Liu Y., Xie J., Wu N., Wang L., Ma Y., Tong J. (2019). Influence of silane treatment on the mechanical, tribological and morphological properties of corn stalk fiber reinforced polymer composites. Tribol. Int..

[25] (2009). Wood-Plastic Composite Flooring. General Administration of Quality Supervision, Inspection and Quarantine of the People’s Republic of China.

[26] Akil H.M., Cheng L.W., Ishak Z.M., Bakar A.A., Abd Rahman M.A. (2009). Water absorption study on pultruded jute fibre reinforced unsaturated polyester composites. Compos. Sci. Technol..

[27] Ma Y., Ma Q.S., Suo J., Chen Z.H. (2008). Low-temperature fabrication and characterization of porous SiC ceramics using silicone resin as binder. Ceram. Int..

[28] (2018). Brake linings for automobiles. General Administration of Quality Supervision, Inspection and Quarantine of the People’s Republic of China.

[29] Ma Y., Liu Y., Wang L., Tong J., Zhuang J., Jia H. (2018). Performance assessment of hybrid fibers reinforced friction composites under dry sliding conditions. Tribol. Int..

[30] Ma Y., Liu Y., Menon C., Tong J. (2015). Evaluation of wear resistance of friction materials prepared by granulation. ACS Appl. Mater. Interfaces.

[31] Satapathy B.K., Bijwe J. (2004). Performance of friction materials based on variation in nature of organic fibres: Part I. Fade and recovery behaviour. Wear.

[32] Nishino T., Hirao K., Kotera M., Nakamae K., Inagaki H. (2003). Kenaf reinforced biodegradable composite. Compos. Sci. Technol..

[33] Taghiyari H.R., Karimi A., Tahir P.M. (2015). Organo-silane compounds in medium density fiberboard: Physical and mechanical properties. J. For. Res..

[34] Siroka B., Noisternig M., Griesser U.J., Bechtold T. (2008). Characterization of cellulosic fibers and fabrics by sorption/desorption. Carbohydr. Res..

[35] Hoikkanen M., Honkanen M., Vippola M., Lepistö T., Vuorinen J. (2011). Effect of silane treatment parameters on the silane layer formation and bonding to thermoplastic urethane. Prog. Org. Coat..

[36] Vilay V., Mariatti M., Taib R.M., Todo M. (2008). Effect of fiber surface treatment and fiber loading on the properties of bagasse fiber–reinforced unsaturated polyester composites. Compos. Sci. Technol..

[37] Petzel E.A., Smart A.J., St-Pierre B., Selman S.L., Bailey E.A., Beck E.E., Walker J.A., Wright C.L., Held J.E., Brake D.W. (2018). Estimates of diet selection in cattle grazing cornstalk residues by measurement of chemical composition and near infrared reflectance spectroscopy of diet samples collected by ruminal evacuation. J. Anim. Sci..

[38] Tonoli G.H.D., Rodrigues Filho U.P., Savastano H., Bras J., Belgacem M.N., Lahr F.R. (2009). Cellulose modified fibres in cement based composites. Compos. Part A.

[39] Yuan J., Zhang Z., Yang M., Guo F., Men X., Liu W. (2017). Surface modification of hybrid-fabric composites with amino silane and polydopamine for enhanced mechanical and tribological behaviors. Tribol. Int..

[40] Karthikeyan S., Rajini N., Jawaid M., Winowlin Jappes J.T., Thariq M.T.H., Siengchin S., Sukumaran J. (2017). A review on tribological properties of natural fiber based sustainable hybrid composite. Proc. Inst. Mech. Eng. Part J.

[41] Elen N.Ç., Yıldırım M., Kanbur Y. (2023). Tribological properties of hemp fiber reinforced polylactic acid bio-composites: Effect of different types of modification methods. Funct. Compos. Struct..

[42] Mylsamy B., Chinnasamy V., Palaniappan S.K., Subramani S.P., Gopalsamy C. (2020). Effect of surface treatment on the tribological properties of Coccinia Indica cellulosic fiber reinforced polymer composites. J. Mater. Res. Technol..

[43] Dharmakrishnan S., Pandian P., Sembian M. (2022). Sustainable characterization of silane treated and untreated Psidium guajava stem natural fibers based automobile brake pads. J. Nat. Fibers.

[44] Wu S., Zhuang J., Wu Q., Qi H., Zhao J., Guo M. (2021). Investigation of tribological, physicomechanical, and morphological properties of resin-based friction materials reinforced with *Agave americana* waste. Mater. Res. Express.

